# Comprehensive analysis of hub genes associated with cisplatin-resistance in ovarian cancer and screening of therapeutic drugs through bioinformatics and experimental validation

**DOI:** 10.1186/s13048-024-01461-w

**Published:** 2024-07-10

**Authors:** Yunshan Zhu, Xuehong Chen, Rongrong Tang, Guangxiao Li, Jianhua Yang, Shihao Hong

**Affiliations:** 1 Department of Obstetrics and Gynecology, Sir Run Run Shaw Hospital, School of Medicine, Zhejiang University, Hangzhou, China; 2 Key Laboratory of Reproductive Dysfunction Management of Zhejiang Province, Hangzhou, 310016 China; 3 Hospital Department of Obstetrics and Gynecology, Linhai Second People’s Hospital, TaiZhou, 317016 China

**Keywords:** Ovarian cancer, Cisplatin resistance, miRNA-mRNA pairs, Molecular docking, Bioinformatics analysis

## Abstract

**Background:**

To identify key genes associated with cisplatin resistance in ovarian cancer, a comprehensive analysis was conducted on three datasets from the GEO database and through experimental validation.

**Methods:**

Gene expression profiles were retrieved from the GEO database. DEGs were identified by comparing gene expression profiles between cisplatin-sensitive and resistant ovarian cancer cell lines. The identified genes were further subjected to GO, KEGG, and PPI network analysis. Potential inhibitors of key genes were identified through methods such as LibDock nuclear molecular docking. In vitro assays and RT-qPCR were performed to assess the expression levels of key genes in ovarian cancer cell lines. The sensitivity of cells to chemotherapy and proliferation of key gene knockout cells were evaluated through CCK8 and Clonogenic assays.

**Results:**

Results showed that 12 genes influenced the chemosensitivity of the ovarian cancer cell line SKOV3, and 9 genes were associated with the prognosis and survival outcomes of ovarian cancer patients. RT-qPCR results revealed NDRG1, CYBRD1, MT2A, CNIH3, DPYSL3, and CARMIL1 were upregulated, whereas ERBB4, ANK3, B2M, LRRTM4, EYA4, and SLIT2 were downregulated in cisplatin-resistant cell lines. NDRG1, CYBRD1, and DPYSL3 knock-down significantly inhibited the proliferation of cisplatin-resistant cell line SKOV3. Finally, photofrin, a small-molecule compound targeting CYBRD1, was identified.

**Conclusion:**

This study reveals changes in the expression level of some genes associated with cisplatin-resistant ovarian cancer. In addition, a new small molecule compound was identified for the treatment of cisplatin-resistant ovarian cancer.

**Supplementary Information:**

The online version contains supplementary material available at 10.1186/s13048-024-01461-w.

## Introduction

Ovarian cancer (OC) remains a prevalent gynecological malignancy despite advancements in diagnosis and treatment over recent decades. This challenge is compounded by the absence of distinctive symptoms, often leading to advanced-stage diagnoses and ranking as the fifth-highest cause of global cancer mortality in women [1, 2]. According to World Health Organization statistics, approximately 150,000 individuals succumb to OC annually worldwide [1–4], with the majority being women over 50 years old [2, 4, 5]. Given the lack of effective early tumor markers and diagnostic methods for ovarian cancer, it is imperative to identify key genes associated with the progression of ovarian cancer, particularly those linked to poor prognosis.

Limited options for early detection and effective treatment are major contributors to poor prognosis and high mortality rates in ovarian cancer. However, chemotherapy resistance has emerged as a critical factor which further hinders successful treatment and worsening patient outcomes [6, 7]. The primary debulking surgery and platinum-based chemotherapy or neoadjuvant chemotherapy, are the first line treatments for ovarian cancer, followed by interval debulking surgery and additional post-surgery chemotherapy [7]. Despite high response rates, the median progression-free survival rate of ovarian cancer patients undergoing these treatments is still low [8]. Furthermore, over 70% of ovarian cancer patients are prone to relapse, developing strong resistance to platinum drugs [9, 10]. Currently, it is challenging to treat ovarian cancer due to the emergence of platinum resistance. Cisplatin, recognized as one of the most effective chemotherapeutic drugs for ovarian cancer, forms DNA-cisplatin crosslinks with tumor cell DNA, inducing DNA damage, inhibiting DNA replication, and promoting cell apoptosis [10–12]. However, tumor cells often acquire drug resistance through various mechanisms [13]. This calls for the development of new molecularly targeted drugs to prevent disease progression. However, the complex mechanisms behind cisplatin resistance and the genetic variability among ovarian cancer patients have limited our ability to alleviate drug resistance [14, 15]. To our knowledge, there are no effective diagnostic markers for predicting cisplatin resistance in ovarian cancer patients. In this study, we aimed to explore the molecular mechanism of cisplatin resistance and develop new targeted therapies against cisplatin resistance in ovarian cancer patients.

## Methods

### Study design and gene expression data collection

In this study, to maximize the generalizability of the cisplatin resistance genes obtained from the final screen, we collected the gene expression profiles of three different cisplatin-resistant/sensitive ovarian cancer cell lines. Three gene expression profiles (GSE33482, GSE45553, and GSE115939) were downloaded from the Gene Expression Omnibus database. Firstly, common differentially expressed genes (DEGs) were screened out. Subsequently, a comprehensive bioinformatics analysis was performed to determine potential molecular mechanisms of drug resistance. Additionally, the hub genes identified were experimentally validated. Finally, we performed virtual screening of FDA-approved drugs and molecular docking of the screened drug-resistant genes to identify compounds with potential inhibitory effects on drug-resistant molecules. GEO database (https://www.ncbi.nlm.nih.gov/geo/) was used to analyze mRNA expression levels in OC patients. The dataset used in our study includes GSE33482, GSE45553and GSE115939.

### Identification of differentially expressed genes (DEGs)

GEO2R was used to identify differential genes [16]. We utilized the GEO2R platform within the GEO database to analyze gene expression differences between cisplatin-resistant and cisplatin-sensitive ovarian cancer patients. Employing default parameters for identifying differentially expressed genes, we downloaded all results in .txt format for further processing using Excel. Genes with *P*-value <0.05 and |log_2_FC|≥1.0 were considered as differential genes. More details in Supplementary File 1. The FunRich tool was used to make Venn diagrams [17].

### Functional analyses

Following the identification of common DEGs, we performed functional enrichment analysis using several databases: GO, KEGG, Reactome, and WikiPathways. These databases were employed to elucidate the biological pathways and functions associated with the common DEGs. *p*<0.05 was considered statistically significant. More details in Supplementary File 2.

### PPI network construction

A PPI network was constructed using STRING database (https://string-db.org) to analyze the interactions between DEGs. If the confidence setting was > 0.7, proteins that do not interact are hidden.

### Prognostic signature of hub genes

Kaplan‐Meier plotter (www. kmplot.com) was used to perform Overall Survival (OS), progression‐free survival (PFS) and Post-Progression Survival (PPS) analyses of hub genes.

### Construction of miRNA-gene network

FUNRICH, miRTarBase [18], Targetscan (v7.0; targetscan.org) and miRDB [19] databases were used to predict miRNA of hub genes.The collected common miRNA and hub genes were connected to construct a miRNA-gene regulatory network. More details in Supplementary File 3.

### Establishment of cisplatin-resistant SKOV3 cells (SKOV3/DDP) and cell culture

The human ovarian cancer cell line SKOV3 (Homo sapiens, human, RRID: CVCL_0532) was purchased from the Cell Bank of the Chinese Academy of Sciences (Shanghai, China). Cisplatin-resistant cell lines were established in our lab. Cell lines were authenticated by STR profiling. All cell lines were cultured in DMEM medium (Hyclone, Logan, U.S.) supplemented with 10% fetal bovine serum (FBS, Gibco) and 100 U/mL of penicillin, and 100 U/mL of streptomycin (Invitrogen, U.S.), and incubated at 5% CO_2_ and 37℃ with saturated humidity. The maintenance concentration of cisplatin in the drug-resistant cell line was 1 μmol/L. All experiments were performed with mycoplasma-free cells.

### RT‐qPCR

Total RNA from SKOV3/DDP cell lines was extracted using the RNA Quick Purification Kit (ES Science, Shanghai, China), and cDNA was synthesized using the cDNA Reverse Transcription kit (Vazyme, Nanjing, China). RT-qPCR was performed using TB Green™ Premix Ex Taq™ II (RR420A; Takara, China) with specific primers on a Bio-Rad CFX-96 Real-time PCR system (Bio-Rad, USA), following the manufacturer’s instructions. The Ct of the identified genes were normalized to GAPDH, an internal control gene, and data were analyzed using the 2^−ΔΔCT^ method. All PCR primers are listed in Supplementary Table 1.

### siRNA transfection

siRNA targeting NDRG1, CYBRD1, and MT2A were designed and synthesized by Ribobio Company (Ribobio, China). SKOV3/DDP cells were seeded into 12-well plates and transfected using lipofectamine 3000 (Invitrogen, Carlsbad, CA, USA) following the manufacturer's protocol. After incubation for 48 hours, the expression levels of NDRG1, CYBRD1, and MT2A were evaluated by qPCR.

### CCK8 Detection and IC50 determination of cisplatin on ovarian cancer cells

Ovarian cancer cells were harvested and seeded into 96-well cell culture plates at a density of 1 × 10^3^ cells/well. The plates were then incubated overnight at 37 °C in a 5% CO_2_ atmosphere. After incubation, 10 μl of CCK8 was added to each well, and the cells were incubated at 37°C for 2 h. The IC50 of cisplatin for both SKOV3 and SKOV3/DDP cell lines was subjected to the CCK8 assay. Cells were treated with various concentrations of cisplatin, ranging from 0, 0.25, 1, 2, 4, 8, 16 and 32 μM. After 48 hours of treatment, CCK-8 was added to each well, and the OD450 value of each well was measured using a spectrophotometer.

### Colony formation

The cell suspension was appropriately diluted and seeded into 6-well plates at a density of 1000 cells per well. The plates were then incubated in a 37°C, 5% CO_2_ environment for 2 weeks. Once colonies were formed, the cells were rinsed twice with PBS, fixed with 4% paraformaldehyde for 10 min, stained with 0.5% crystal violet for 30 min, and subsequently counted and photographed under an optical microscope.

### Molecular docking

Molecular docking was conducted using the Discovery Studio, a widely used software for molecular modeling, virtual screening, and molecular simulation. The software shortens the time and cost associated with screening potential drugs for diseases. Protein crystal structures were obtained from the PDB data and preprocessed using the prepared protein module in Discovery Studio 2019. The dataset used for virtual screening was obtained from The NCGC Pharmaceutical Collection database [20], comprising drugs approved by the FDA. Docking experiments were conducted using LibDock, and the ligand pose with the highest score was considered the optimal docking pose.

### Statistical analysis

GraphPad Prism 8.0 was utilized for plotting and analyzing IC50 values and other data, with all results presented as Mean ± SEM. Differences between groups were analyzed using the student’s t-test and *P* < 0.05 was considered statistically significant.

## Results

### Identification of DEGs between cisplatin-resistant and sensitive ovarian *cancer* cell lines

The gene expression profiles of cisplatin-resistant ovarian cancer cell lines (GSE33482, GSE45553, and GSE115939) were obtained from the GEO database. The datasets selected encompassed diverse ovarian cancer cell lines, GSE33482 included gene expression data from a cisplatin-sensitive/resistant ovarian cancer cell line (A2780); GSE45553 comprised 4 cases of cisplatin-sensitive and 4 cases of cisplatin-resistant cell lines, while GSE115939 involved two experimental groups, IGROV1 wild-type cells, and IGROV1 Cisplatin- resistant cells, each with two biological replicate samples.

**Fig. 1:**
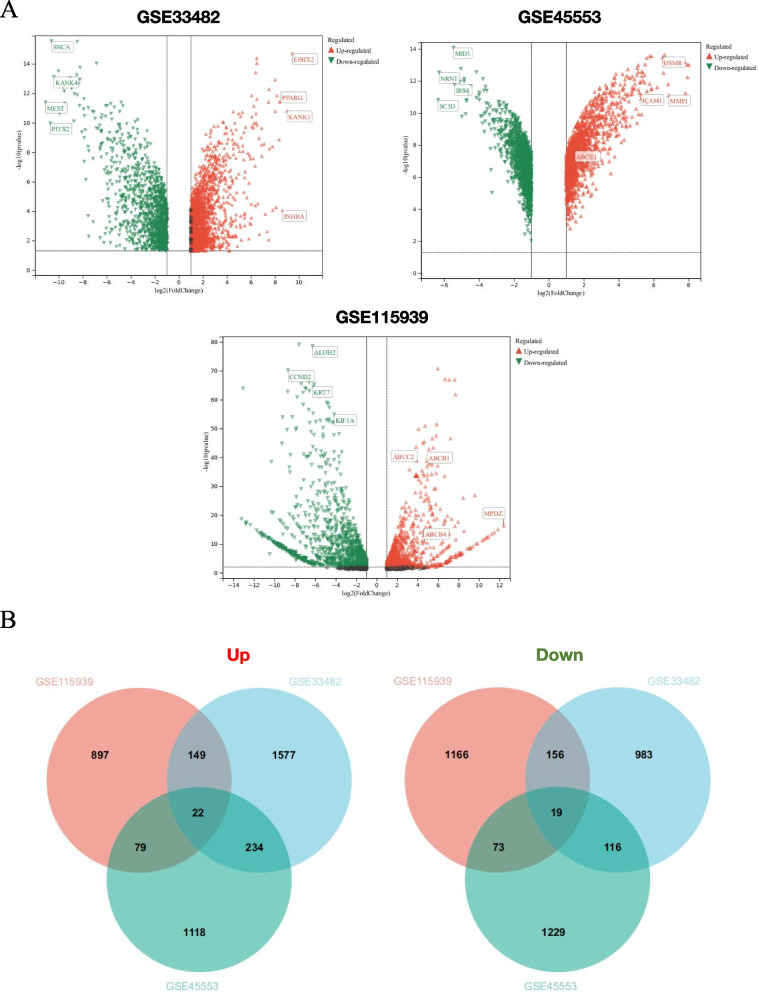
DEGs analysis. **A** DEGs between the cisplatin-sensitive and cisplatin-resistant groups are shown on a volcano plot. **B** Venn diagram shows common DEGs

To ensure the reproducibility of subsequent research, GEO2R was directly employed to analyze the DEGs in all data sets. The threshold was set at a *p*-value < 0.05, and a fold change ≥ 2. The volcano plots for the three datasets (GSE33482, GSE45553, and GSE115939) depicting the DEGs are presented (Fig. 1A). Furthermore, detailed expression information for all genes can be accessed in Supplementary File 1. The Venn diagram illustrates a total of 41 DEGs with consistent expression trends identified from the three datasets, comprising 22 commonly up-regulated and 19 commonly down-regulated genes (Fig. 1B).

### Functional enrichment of common DEGs and construction of protein-protein interaction networks

**Fig. 2:**
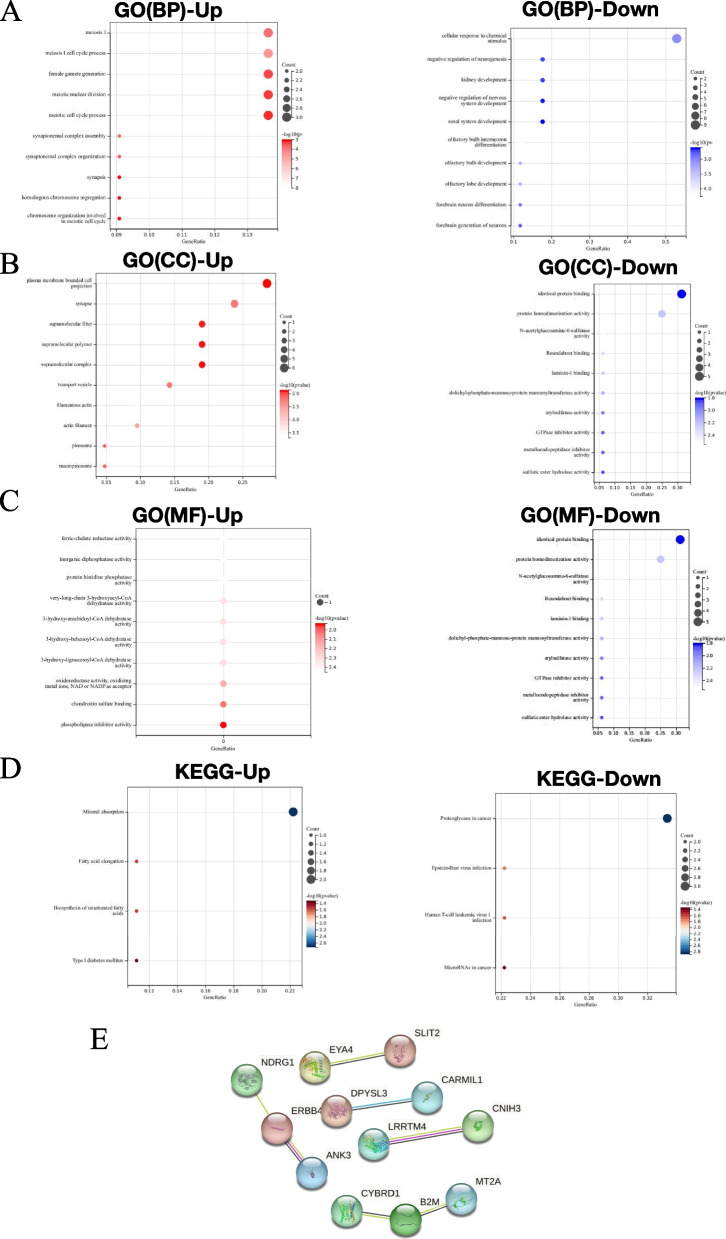
Functional analysis. **A** GO terms (Biological Process, BP). **B** GO terms (Cellular Component, CC). **C** GO terms (Molecular Function, MF). **D** KEGG Enrichment. **E** DEGs were filtered into the PPI network through STRING database

The 22 co-upregulated and 19 co-downregulated genes were analyzed using the GO database, including biological process (BP), cellular component (CC), and molecular function (MF). Applying a *p*-value <0.05 as the threshold, the genes were mapped to the background set (Fig. 2A). In the GO-BP classification, the up-regulated DEGs were significantly involved in cell division and cell cycle progression, while down-regulated DEGs were significantly involved in neuronal differentiation, organ development, and related processes. For GO-CC classification, upregulated DEGs were associated with filamentous actin, actin filaments, synapses, transport vesicles, and chimeras, whereas downregulated DEGs were associated with synapses, basement membranes, MHC class I complexes, and the extracellular matrix, etc. (Fig. 2B). In GO-MF classification, upregulated DEGs were enriched for activities such as ferric-chelate reductase, inorganic diphosphatase, protein histidine phosphatase, very-long-chain 3-hydroxyacyl-CoA dehydratase, and 3-hydroxy-arachidoyl-CoA dehydratase. Conversely, down-regulated DEGs were enriched for identical protein binding, protein homodimerization activity, N-acetylglucosamine-6-sulfatase, Roundabout binding, and laminin-1 binding (Fig. 2C). The KEGG database was also employed to explore the potential biological functions of DEGs in drug resistance. Fig. 2D shows that up-regulated DEGs were involved in important pathways including proteoglycans in cancer, epstein-Barr virus infection, Human T-cell leukemia virus 1 Infection, and microRNAs in cancer, while down-regulated DEGs participated in important pathways including mineral absorption, fatty acid elongation, biosynthesis of unsaturated fatty acids, and type I diabetes mellitus. Specific details of functional enrichment analysis can be found in the Supplementary file 2.

PPI networks of the 41 DEGs were constructed and visualized using the STRING database. After removing isolated nodes, the remaining DEGs formed a complex multi-center interaction network graph, comprising 12 nodes (up-regulated genes: NDRG1, CYBRD1, MT2A, CNIH3, DPYSL3, CARMIL1; down-regulated genes: ERBB4, ANK3, B2M, LRRTM4, EYA4, SLIT2) and 7 edges (Fig. 2E).

### Prognostic survival analysis

**Fig. 3:**
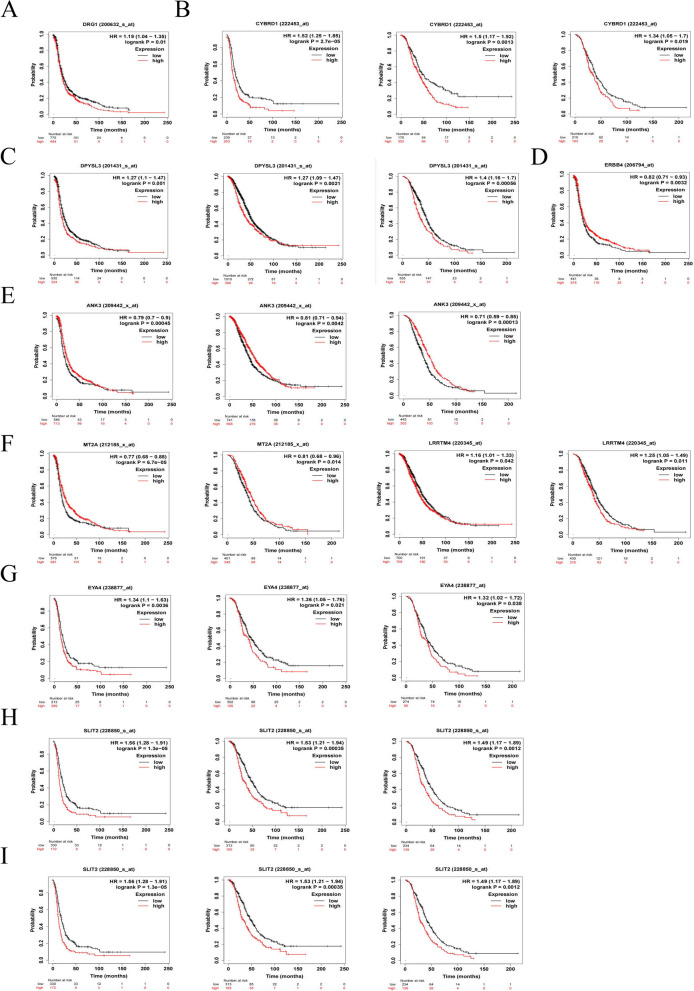
Prognostic survival analysis. **A** OS of NDRG1. **B** PFS, OS and PPS of CYBRD1. **C** PFS of DYBRD1. **D** PFS of ERBB4. **E** PFS, OS and PPS of ANK3. **F** PFS and PPS of MT2A. **G** OS and PPS of LRRTM4. **H** PFS, OS and PPS of EYA4. **I** PFS, OS and PPS of SLIT2

The prognostic significance of the 12 hub genes was examined through Kaplan-Meier analysis, focusing specifically on patients who had undergone platinum-based drug treatment. In the platinum-based treatment cohort, except for B2M, CNIH3, and CARMIL1, the remaining 9 genes demonstrated a close association with patient prognosis and survival (Fig. 3). High expression of NDRG1 correlated with poorer PFS in patients (Fig. 3A). High expression of CYBRD1 (Fig. 3B) and DPYSL3 (Fig. 3C) was associated with poorer PFS, OS, and PPS in cisplatin-resistant patients. Reduced expression of ERBB4 was associated with poorer PFS in cisplatin-resistant patients (Fig. 3D). Low expression of ANK3 was associated with poorer PFS, OS, and PPS in cisplatin-resistant patients (Fig. 3E). Notably, high expression of MT2A was associated with improved PFS and PPS in cisplatin-resistant patients (Fig. 3F). Low expression of LRRTM4 was associated with better OS and PPS in cisplatin-resistant patients (Fig. 3G). Reduced expression of EYA4 (Fig. 3H), and SLIT2 (Fig. 3I) was associated with improved PFS, OS, and PPS in cisplatin-resistant patients.

**Fig. 4:**
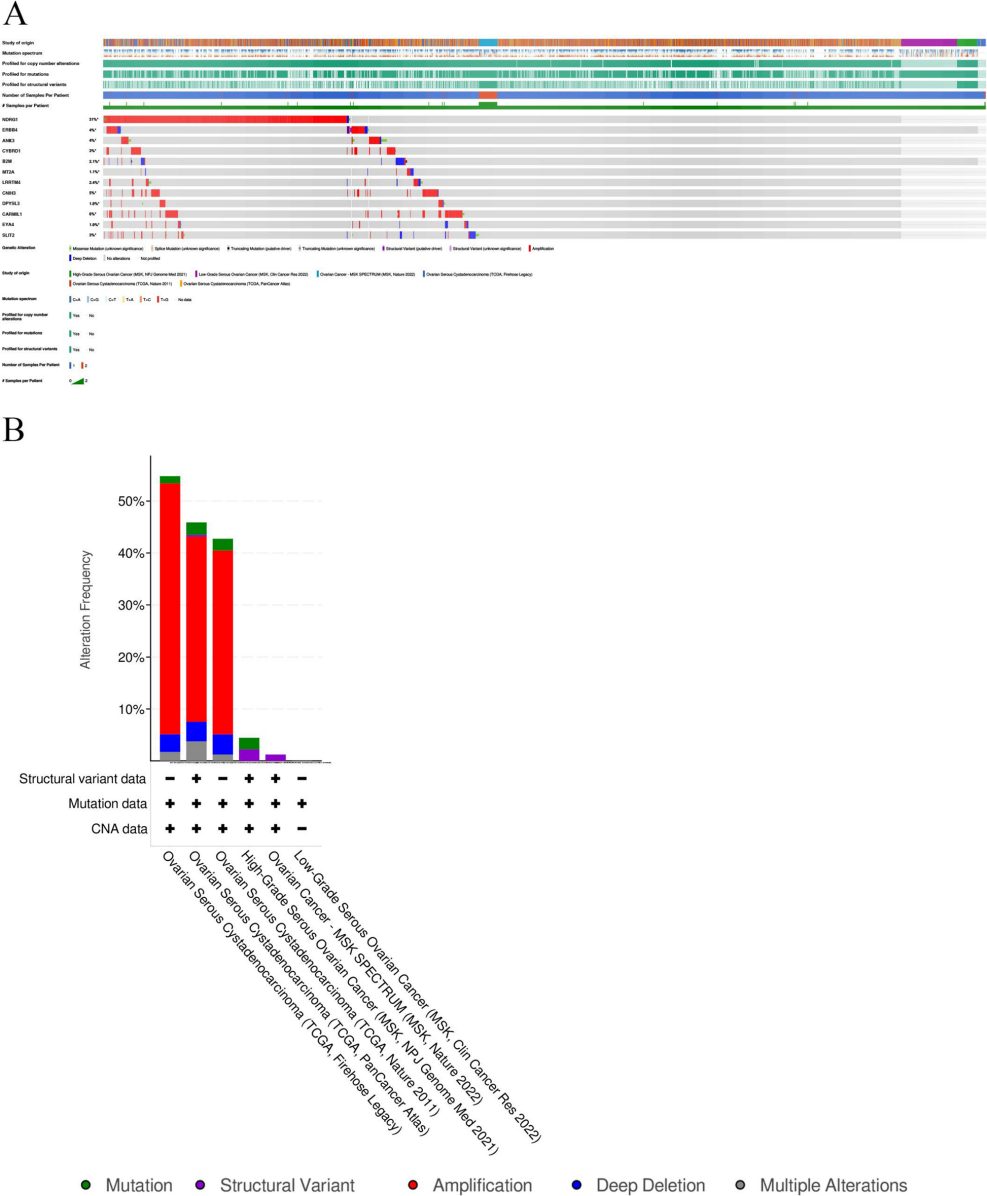
Information on the genetic alterations of the hub genes. **A** The genetic alterations related to the hub genes are shown through a visual summary across a set of ovarian serous cystadenocarcinoma samples. **B** An overview of the alterations of hub genes in the genomics datasets of ovarian serous cystadenocarcinoma in the TCGA database

Furthermore, genetic alteration information for these key genes was analyzed using cBioPortal. As shown in Fig. 4, the 12 hub genes exhibited varying degrees of genetic alterations in 1880 patients with serous ovarian cancer. These changes included missense mutations, structural variants, splicing mutations, amplifications, deep deletions, etc. (Fig. 4A). Notably, NDRG1 was amplified in 31% of these 1880 patients, with gene amplification being the predominant alteration in different types of serous ovarian cancer (Fig. 4B).

### Construction of miRNA-hub gene regulatory network

**Fig. 5:**
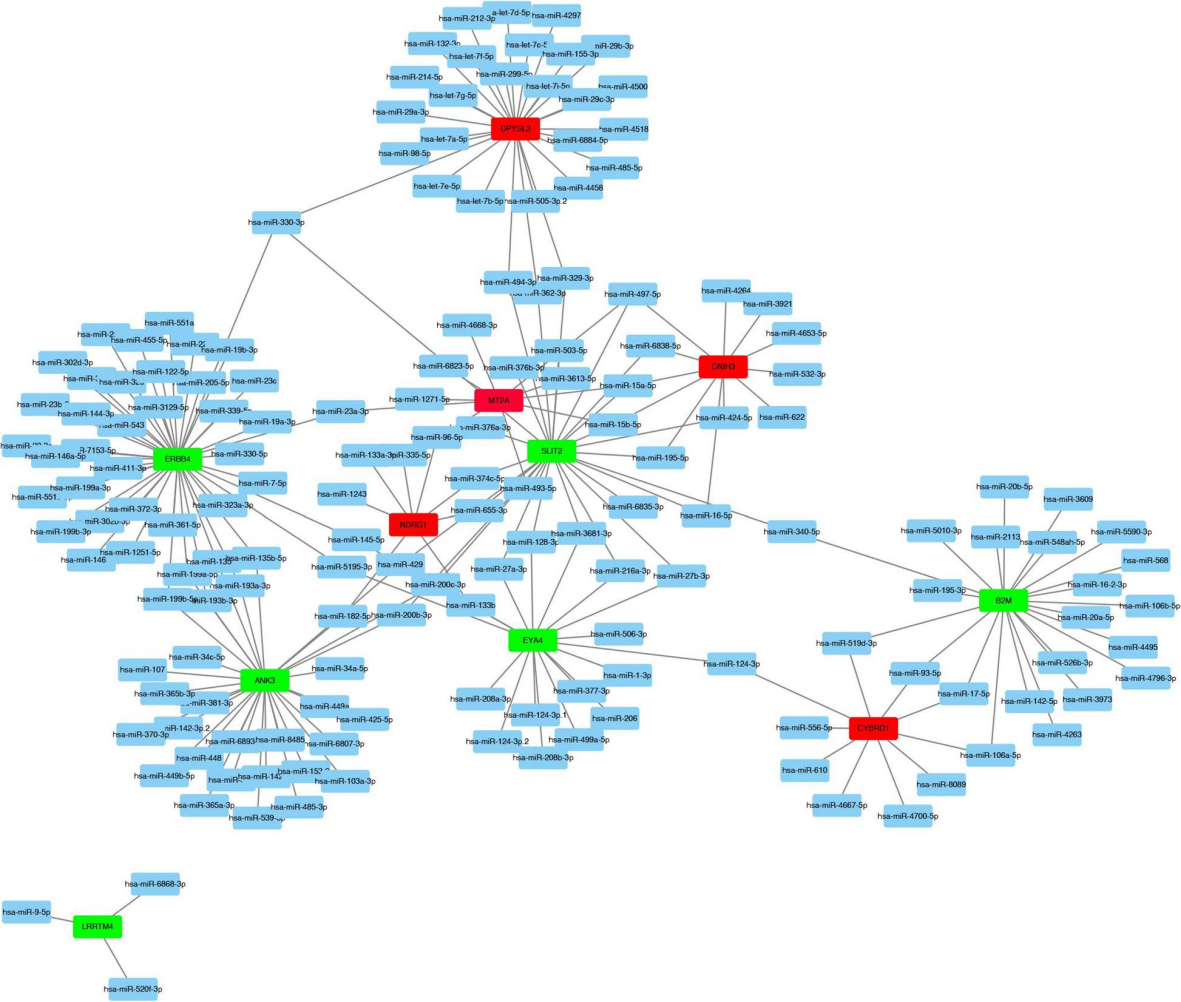
Construction of miRNA-hub gene regulatory network. The collected common miRNA and hub genes were connected to construct a miRNA-gene regulatory network

To better understand the potential mechanisms of hub genes in cisplatin regulation in ovarian cancer patients, gene regulatory networks were analyzed. Only miRNAs present in at least two databases (FUNRICH, miRTarBase, Targetscan, miRDB) are considered target miRNAs of hub genes. The Cytoscape software was used to map the miRNA-hub gene regulatory network. As shown in Fig. 5, the gene regulatory network consisted of 11 hub genes and 211 miRNAs (Supplementary file 3). Notably, the miRNAs appearing in all four databases included: hsa-miR-182-5p (target miRNA of NDRG1); hsa-miR-17-5p, hsa-miR-20a-5p, hsa-miR-93 -5p, hsa-miR-106b-5p, hsa-miR-20b-5p, hsa-miR-519d-3p (miRNA targeting CYBRD1); hsa-miR-27b-3p (miRNA targeting EYA4); hsa-miR-221-3p (miRNA targeting ERBB4); and hsa-miR-340-5p (miRNA targeting B2M). Additionally, hsa-miR-330-3p, hsa-miR-497-5p, hsa-miR-15a-5p, hsa-miR-15b-5p, and other miRNAs jointly regulate multiple hub genes.

### In vitro validation of the role of 12 genes in cisplatin resistance

**Fig. 6:**
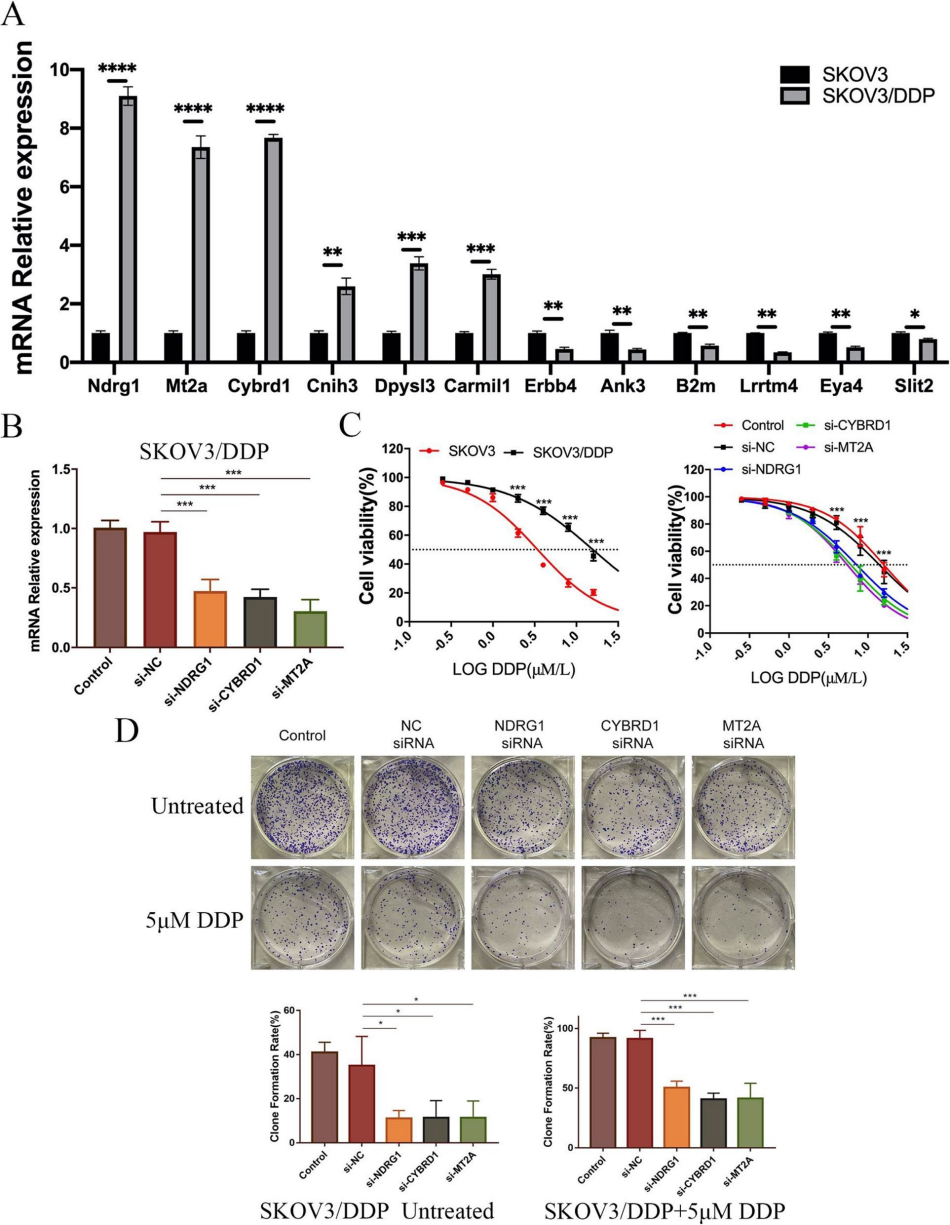
In vitro validation of hub genes. **A** 14 genes in SKOV3 cells and SKOV3/DDP cells were measured by qRT‐PCR. **B** The expression of NDRG1, CYBRD1 and MT2A genes detected in SKOV3/DDP cells by qRT‐PCR. **C** Left panel: The cell viability of SKOV3 and SKOV3/DDP cells was assessed using CCK-8 assay at 48 hours.The IC50s of DDP for SKOV3 and SKOV3/DDP cells were 3.431 and 15.76 μM/L, respectively. Right panel: The cell viability of SKOV3/DDP cells and 3 gene knockdown SKOV3/DDP cell lines were assessed using CCK-8 assay at 48 hours **D** Colony formation of untreated SKOV3/DDP cells and SKOV3/DDP cells+5 μmol DDP and statistical analysis from 3 independent experiments .(****P < 0.0001; ***P < 0.001; **P < 0.01;*P < 0.05, Student’s t-test, Error bars are±SEM)

To validate the results of the bioinformatics analysis, cisplatin-resistant SKOV3 cell lines were obtained through cisplatin-pressurized screening. Gene expression levels were detected by qPCR, indicating upregulation of NDRG1, CYBRD1, MT2A, CNIH3, DPYSL3, and CARMIL1, while ERBB4, ANK3, B2M, LRRTM4, EYA4, and SLIT2 were downregulated (Fig. 6A). Subsequently, three 3 significantly upregulated genes NDRG1, CYBRD1, and MT2A were selected for further study. Cisplatin-resistant SKOV3 cell lines with knockdown of NDRG1, CYBRD1, and MT2A were constructed using siRNA, and knockdown efficiency was verified by qPCR (Fig. 6B). CCK8 (Fig. 6C) and clonogenic assays (Fig. 6D) were performed with SKOV3 cells and cisplatin-resistant SKOV3 cells. Compared to these control cells, knocking down NDRG1, CYBRD1, and MT2A significantly reduced the ability of the cells to proliferate and form colonies, even in the absence of cisplatin treatment.

### Small molecule compound screening

**Fig. 7:**
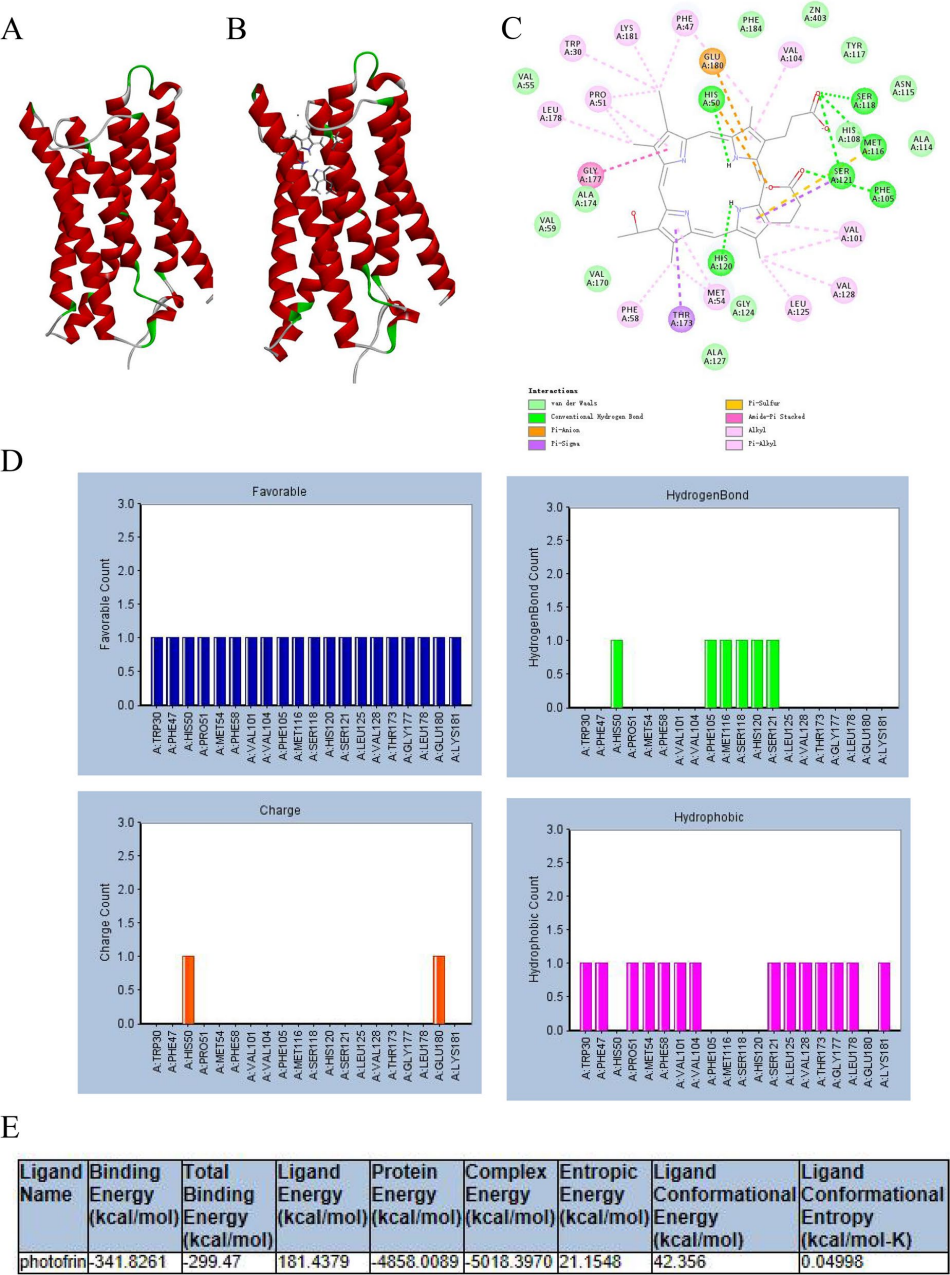
Virtual screening of small molecules inhibitors. **A** The crystal structure of CYBRD1. **B** the predicted binding modes of photofrin with CYBRD1. **C** The docking detail between photofrin with CYBRD1. **D** Analyze thebond-forming relationships between photofrin and CYBRD1. **E** Calculate the binding free energy for the docked pose

Based on the results of survival analysis and existing research, CYBRD1 was selected as the target for drug screening. The 3D structure of CYBRD1 (PDB: 5ZLG) was obtained from the PDB database (Fig. 7A). Protein-drug virtual screening was conducted through the LibDock program, revealing the best docking posture shown in Fig. 7B. The results indicated that CYBRD1 and photofrin had the highest binding scores. Furthermore, hydrogen bond interactions were analyzed (Fig. 7C), demonstrating that CYBRD1 formed 7 pairs of hydrogen bonds with photofrin. Finally, the binding free energy of CYBRD1 and photofrin was calculated (Fig. 7D). When the value of binding free energy is negative, the system is stable.

## Discussion

Ovarian cancer is the leading cause of mortality among women, with the worst prognosis among gynecological tumors [1, 2, 4]. Over the last few decades, chemotherapy has shown promising results in ovarian cancer patients, with cisplatin therapy emerging as the primary treatment option [9–12]. While most ovarian cancer patients initially respond well to cisplatin treatment, resistance inevitably develops over time [9–12]. This emerging resistance presents a major hurdle in treating ovarian cancer, currently standing as the biggest obstacle doctors face in the clinic. To unravel the complex mechanisms behind this resistance, researchers are increasingly turning to big data analysis. Currently, bioinformatics analysis is widely used to analyze diagnostic and therapeutic targets for various cancers [21–24]. In this study, to identify potential therapeutic targets for cisplatin resistance in ovarian cancer patients, data were obtained from three GEO datasets. Each dataset was derived from a cisplatin-resistant/sensitive ovarian cancer cell line. The rationale behind selecting samples from different cisplatin-resistant/sensitive cell lines lied in the need to expand the sample size and enhance statistical robustness. In total, 10 cisplatin-sensitive and 10 cisplatin-resistant cell line samples were included in this study. Differential expression analysis identified 41 common DEGs which were presented in Venn diagrams, comprising 22 upregulated and 19 downregulated genes. Subsequently, GO and KEGG analyses were conducted to identify the biological functions of these DEGs and mechanisms behind cisplatin resistance. A PPI network was constructed to investigate the shared regulatory pathways between these genes. The prognostic value of these DEGs was determined through survival analysis. Bioinformatic analysis revealed that NDRG1, CYBRD1, and MT2A expressions upregulated and were associated with poor prognosis of ovarian cancer patients in the platinum-based treatment group, whereas upregulation of ERBB4 and ANK3 correlated with improved prognosis. Our research revealed some interesting paradoxes. For instance, a gene called MT2A showed significantly higher expression in the cisplatin-resistant group. However, patients within the cisplatin-treated group who had high MT2A expression actually had a better prognosis. Similarly, three other genes (LRRTM4, EYA4, and SLIT2) were expressed at much lower levels in the cisplatin-resistant group. Surprisingly, low expression of these genes was also linked to a better prognosis for patients. These findings highlight the intricate nature of gene regulation and the multifaceted mechanisms underlying drug resistance. Although big data offers a powerful tool for analyzing drug-resistant genes, interpreting the results requires a cautious approach. The intricate ways genes are regulated and the multifaceted mechanisms of drug resistance can lead to seemingly contradictory findings. Researchers should carefully consider these complexities to draw accurate conclusions from big data analysis.

In this study, we further explored the upregulated genes associated with poor prognosis in ovarian cancer patients in the cisplatin-resistant group. NDRG1, a gene expressed in various tumors, has been reported to have pleiotropic effects in cancer [25]. Luo et al. discovered a strong correlation between high NDRG1 expression and metastasis and recurrence of breast cancer. Nagai et al identified NDRG1 as an independent prognostic factor for breast cancer [26]. NDRGl's role in cancer appears complex and context-dependent. Studies have shown it plays a crucial role in regulating the progression and chemoresistance of triple-negative breast cancer [27]. Conversely, NDRG1 appears to act as a tumor suppressor in prostate and pancreatic cancers [28]. Interestingly, a large-scale analysis of mutations in women's cancers (breast, ovarian, endometrial, and cervical) revealed a high mutation frequency (19%) in NDRG1, second only to MYC (22%). This suggests a potential role for NDRG1 in these cancers, particularly those influenced by hormones [29]. This suggests that NDRG1 may still have undiscovered roles in ovarian cancer. CYBRD1 functions as a ferric iron reductase, regulating signaling pathways related to iron metabolism [30]. Chen et al. used big data to show that CYBRD1 as an independent predictor of adverse outcomes in ovarian cancer can be used in predicting clinical prognosis [31]. Additionally, a meta-analysis of single-gene prognostic biomarkers in ovarian cancer revealed a correlation between high CYBRD1 expression and poor prognosis [32]. Although few studies have documented the association of CYBRD1 with ovarian cancer, the evidence suggests that CYBRD1 can treat ovarian cancer. DPYSL’s involvement in cancer is less explored, with current findings linking it solely to the metastasis of lung cancer [33], prostate cancer [34], breast cancer [30], and liver cancer [35].

MT2A has been implicated in the development of various cancers [36–38]. Zhao et al. showed that MT2A confers oxaliplatin resistance in colorectal cancer cells [38]. Shimizu M et al found that MT2A exacerbates ESCC progression [37]. Another study on high-grade epithelial ovarian cancer identified that MT2A expression was increased after chemotherapy, highlighting its potential association with drug resistance [36]. ERBB4, a member of the ErbB/HER family, has been detected in malignant tumors [39]. In contrast to its family members, EGFR and ERBB2, the role of ERBB4 in human malignancies is relatively ambiguous, displaying dual identities as both a tumor suppressor protein and an oncoprotein [40, 41]. Currently, the role of ERBB4 in the regulation of ovarian cancer cell growth is controversial. While some studies suggest that ERBB4 is a poor prognostic factor in ovarian cancer, others indicate that is inhibits cancer growth [39]. Notably, research on ERBB4 in ovarian cancer primarily focuses on benign ovarian tissue and malignant ovarian tumors. SLIT2 is a tumor suppressor in ovarian cancer [42, 43]. Qiu et al. found that the SLIT2 promoter was significantly hypermethylated in ovarian cancer samples [43]. Lin et al. demonstrated that SLIT2 knockdown in an ovarian cancer cell model increased cell migration and enhanced the expression of multiple oncogenic signaling pathways [42]. Moreover, our study employed Discovery Studio for the virtual screening of drugs targeting the CYBRD1 protein. The selected drugs are all FDA-approved drugs (~1500 compounds)with good safety profiles, which reduces the time and cost associated with new drug development. The Libdock program assigns scores to each docking pose, with a higher score indicating a more stable docking pose. The results indicated that photofrin can bind with CYBRD1 with high affinity, demonstrating interaction potential. This suggests its potential use as a targeted drug for cisplatin-resistant ovarian cancer patients.

In this study, multiple cisplatin resistance datasets were used to identify key genes associated with cisplatin resistance in ovarian cancer, making the screened genes universally relevant. In addition, *in vitro* experiments showed that NDRG1, CYBRD1, and MT2A played a role in ovarian cancer cisplatin resistance. Our study also presents some exciting opportunities for future research. By utilizing virtual screening technology, we identified potential drugs targeting the newly discovered resistance genes. This approach has the potential to significantly reduce the time and cost associated with traditional drug discovery methods. However, it's important to acknowledge the limitations of this initial study. Firstly, the relatively small sample size might have limited our ability to identify all relevant cisplatin-resistance genes. Secondly, we haven't fully explored the specific mechanisms by which these genes contribute to drug resistance. In future, research should focus on two key areas: 1) elucidating the detailed mechanisms of resistance mediated by these genes, and 2) validating and developing the potential drugs identified through virtual screening.

## Conclusion

In summary, this study explores key genes associated with cisplatin-resistant ovarian cancer. The results demonstrate that NDRG1, CYBRD1, MT2A, DPYSL3, ERBB4, ANK3, LRRTM4, EYA4, and SLIT2 can serve as predictive biomarkers for both cisplatin resistance and poor prognosis in OC patients. Notably, cell experiments were performed to validate the roles of NDRG1, CYBRD1, and MT2A in cisplatin resistance in ovarian cancer. Additionally, the study identifies photofrin as a potential CYBRD1-targeted drug for cisplatin-resistant ovarian cancer patients. These findings present a promising target for drug development to overcome cisplatin resistance in OC.

### Supplementary Information

Supplementary Material 1.

Supplementary Material 2.

Supplementary Material 3.

Supplementary Material 4.

## Data Availability

The datasets used in this study, GSE33482, GSE45553and GSE115939, were downloaded from GEO database. ( https://www.ncbi.nlm.nih.gov/geo/query/acc.cgi?).
