# Supplementary material for: Comprehensive analysis of hub genes associated with cisplatin-resistance in ovarian cancer and screening of therapeutic drugs through bioinformatics and experimental validation
Source: J Ovarian Res. 2024 Jul 10;17:142. doi: 10.1186/s13048-024-01461-w (PMC11234624; doi:10.1186/s13048-024-01461-w)
Supplement: Supplementary file 4 — Supplementary Material 4. [file 13048_2024_1461_MOESM4_ESM.docx]

**Supplementary** **Table 1** Sequences of primers used for qRT-PCR analysis

| Name | Sequences |  | Product size(bp) |
| --- | --- | --- | --- |
| NDRG1 | F :5' CCAACAAAGACCACTCTCCTC 3' | R :5' CCATGCCCTGCACGAAGTA 3' | 106 |
| CYBRD1 | F :5' GCTCCGCTTTCTCTCCGAG 3' | R :5' TGTCAATCCCATAAGTGCTGTTG 3' | 99 |
| MT2A | F: 5' CGGAGTCTTCGGATAAGCTCT 3' | R: 5' TTTCCATCAAACATGGGCGAC3' | 136 |
| CNIH3 | F: 5' TAGCTCAGAACTAGCCTACGAC3' | R: 5' GAAGGCCAGCTTACACCAGG 3' | 99 |
| DPYSL3 | F: 5' CCTCGGCATAGATGGAACCC 3' | R:5' CAGCAAGGAGTTGATGTAGTCC3' | 100 |
| CARMIL1 | F: 5' GAGATTCATGGCGTCGTTTGC 3' | R:5' CACCTCACTCACGTCCTCG 3' | 105 |
| ERBB4 | F:5' GCAGATGCTACGGACCTTACG3' | R: 5' GACACTGAGTAACACATGCTCC 3' | 132 |
| ANK3  B2M  LRRTM4  SLIT2  EYA4  GAPDH | F: 5' GAAGATGCAATGACCGGGGA 3'  F: 5' GAGGCTATCCAGCGTACTCCA 3'  F: 5'TTTTCCACGTCTCTTCAACCTC3'    F: 5'AGCTTAGACGAATTGACCTGAGC 3'  F: 5'AGACTCAGTATTCGGGGATGC 3'  F: 5'CTGGGCTACACTGAGCACC 3' | R:5' CTAAAGCCCATGTAACCCTCTG 3'  R:5' CGGCAGGCATACTCATCTTTT 3'  R:5' CGGCTCAATTCCTTGGATGTC 3'  R:5' CCGAAGGCAGTTTATCTTGTTGG 3'  R:5' CCCAAATCGTAAGTGGGCAAG3'  R:5' AAGTGGTCGTTGAGGGCAATG 3' | 101  248  233  191  91  101 |
